# A 54-year follow-up in a woman with pacemaker dependence

**DOI:** 10.1093/ehjcr/ytae589

**Published:** 2024-10-30

**Authors:** Huaner Ni, Fang Wang

**Affiliations:** Department of Cardiology, Shanghai General Hospital, Shanghai Jiao Tong University School of Medicine, Shanghai 200080, China; Department of Cardiology, Shanghai General Hospital, Shanghai Jiao Tong University School of Medicine, Shanghai 200080, China

## Case description

**Figure 1:**
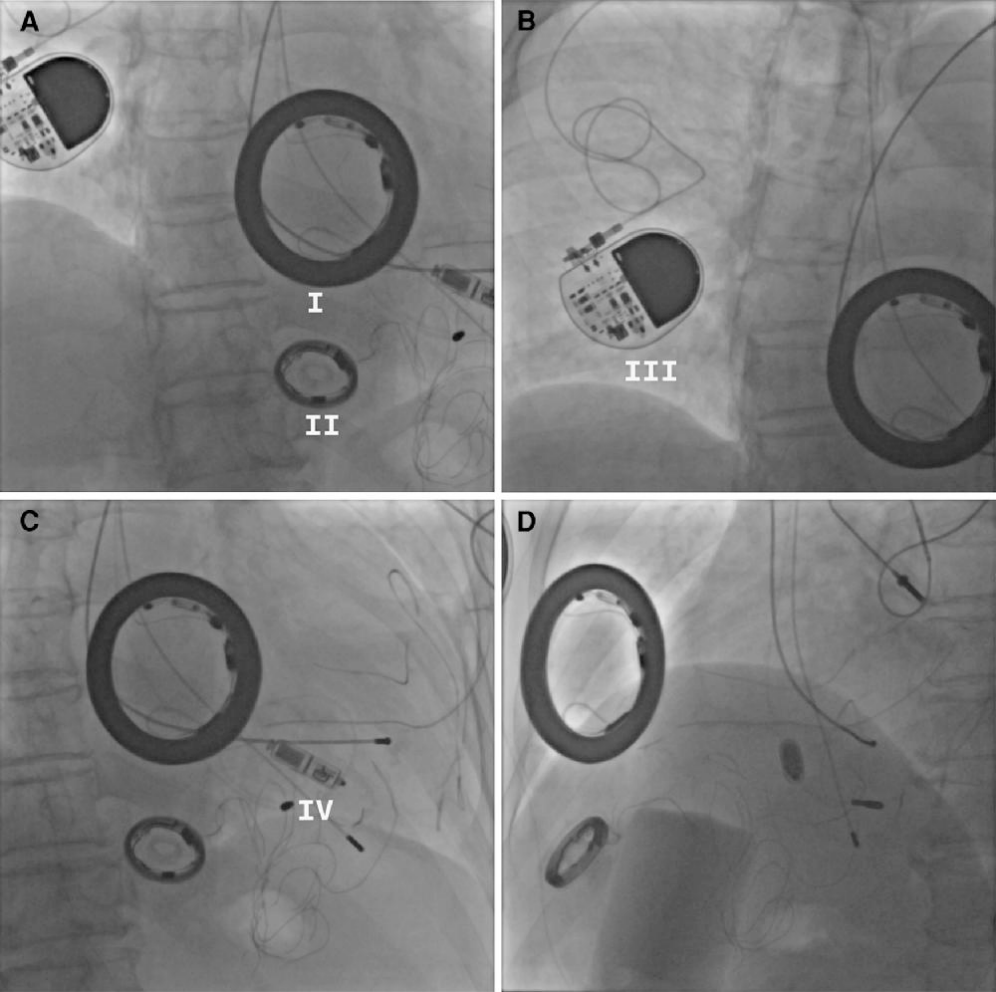
(*A*) Thick-film circuit (I) and radiofrequency inductor coil (II). (*B*) Medtronic pacemaker (III). (*C*) Micra leadless pacemaker (IV). (*D*) Lateral view of (*C*).

In 1970, a 27-year-old woman presented with viral myocarditis and third-degree atrioventricular block, with an electrocardiogram showing a heart rate of 23 b.p.m. Her severely bradycardic condition led to frequent syncopal episodes. Given the limited therapeutic options at the time, she was bedridden for an extended period, with life support maintained by intravenous isoprenaline infusion. On 10 May 1971, she underwent implantation of a self-designed inductive semi-implanted pacemaker via traditional thoracotomy. However, the device malfunctioned within hours due to a pulse sensor failure. Isoprenaline therapy was resumed while she awaited a second pacemaker. In June 1972, a novel pacemaker incorporating a thick-film circuit (*Figure 1A*, I) and a radiofrequency inductor coil (*Figure 1A*, II) was implanted. This device failed after 10 days due to lead fracture. Later in 1972, she received an implantable pacemaker from Siemens-Elema (Sweden), which functioned steadily for 2 years. From 1974 to 1982, she was implanted with domestic pacemakers, necessitating 10 replacements due to issues such as electrode corrosion, screw rusting, infections, and early battery depletion. Since 1982, pacemaker reliability has significantly improved, with device longevity extending to 5 years.^1,2^ The rapid advancement of pacemaker technology allowed her to gradually resume normal life and work. In December 2018 and 2023, she received her 22nd Medtronic pacemaker (*Figure 1B*, III) and her 23rd Micra leadless pacemaker (*Figure 1C*, IV, and *D*), respectively. Now, at 80 years old, she holds the record as the longest-surviving woman with pacemaker dependence, spanning 53 years.

**Consent:** The authors confirm that written consent for submission and publication of this case has been obtained from the patient in line with the Committee on Publication Ethics (COPE) guidelines.

**Funding:** This work was supported by the National Key Research and Development Program of China (number 2023YFB3208200), National Natural Science Function of China (82200488, 82370318, and 82070334), and Shanghai Pujiang Programme (23PJD081).

## Data Availability

The original data are available from the authors upon reasonable request.
